# Supplementary material for: Sugar Transporter, CmSWEET17, Promotes Bud Outgrowth in Chrysanthemum Morifolium
Source: Genes (Basel). 2019 Dec 24;11(1):26. doi: 10.3390/genes11010026 (PMC7017157; doi:10.3390/genes11010026)
Supplement: Supplementary file 1 [file genes-11-00026-s001.pdf]

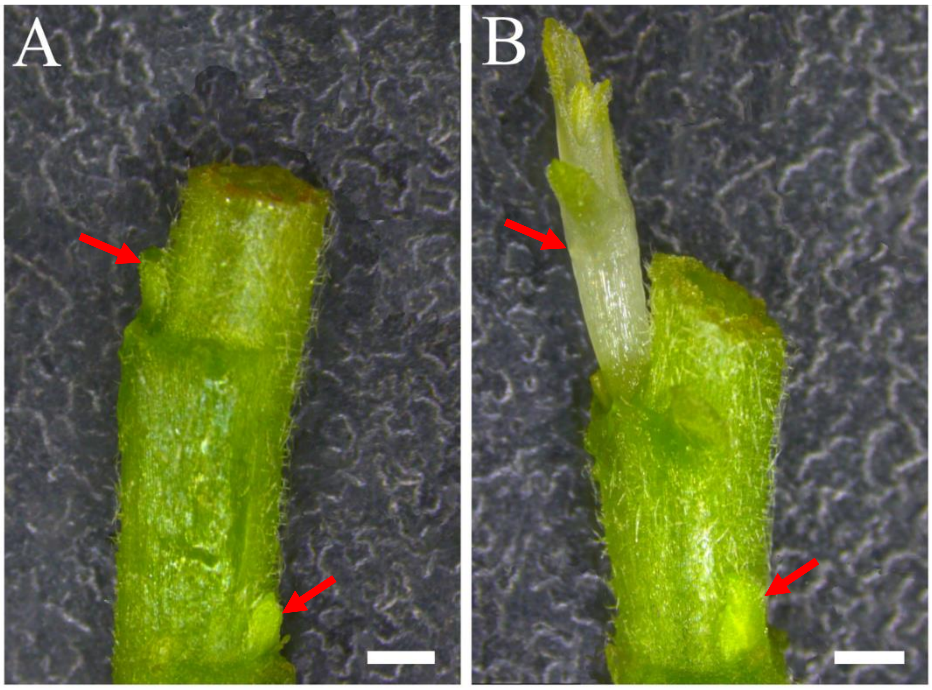

Supplementary Figure 1. Sucrose promotes axillary bud growth in *Chrysanthemum morifolium.* (A) 0 mM sucrose treatment; (B) 20 mM sucrose treatment. Scale bars: 1 mm; red arrows: axillary buds.

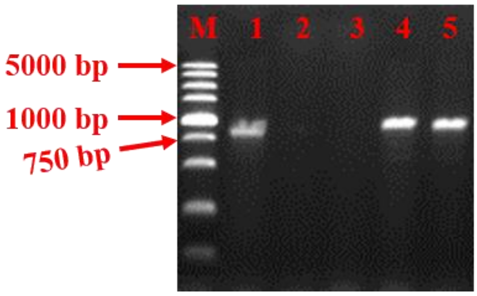

Supplementary Figure 2. Gel electrophoresis with the products of PCR analysis of genomic DNA (primers pMDC43-F and CmSWEET17-R). M: DNA Marker 5000; 1: plasmid pMDC43-*CmSWEET17*; 2: H_2O; 3: Wild-type; 4: OX-39; 5: OX-40.

Supplementary Table 1. Sequences of primers used in the present study.

Primer name	Sequence (5' to 3')
CmSWEET17-F	ATGGCTACCACCATTAGCTTC
CmSWEET17-R	TCATATCTTTTCAATCGACG
CmSWEET17-1A-F	TTCAGTCGACATGGCTACCACCATTAGCTT
CmSWEET17-1A-R	GAGTGCGGCCGCGATATCTTTTCAATCGACG
pMDC43-F	TTACCTGTCCACACAATCTGCC
CmSUC2-Q-F	GGTGTCTCCACCGGTGACA
CmSUC2-Q-R	AATACCTATGGCGCGTGGTT
CmSUC3-Q-F	GGGCATGCTTTCTCGTCATT
CmSUC3-Q-R	AAGGTTGAACCACAAGACCTGTAAT
CmSUC4-Q-F	GCATGCCGTTAGCTGTTACG
CmSUC4-Q-R	ACTCAATCCGTGAGGCTACCA
CmHXK1-Q-F	AAACAATCGTGGAGGTATGTGAC
CmHXK1-Q-R	TCCGGCAGCCGAGAGA
CmHXK2-Q-F	GCGGGTGATAGACACCGAAT
CmHXK2-Q-R	ACGTGCCTGACATCAAGTCTTG
CmHXK3-Q-F	TGAGCGAGCTGATGCAGTAATA
CmHXK3-Q-R	TTGATAACCATGCCTCCTGAAGT
CmSnRK1-Q-F	TGAAATGCAGGTGGGTTCCT
CmSnRK1-Q-R	GAGCTCATGGCACCATCATTC
CmTOR-Q-F	CATTCTCCATGTCGCTCGAA
CmTOR-Q-R	TGAAGAAATTGAGCGGAAGATG
CmSWEET1-Q-F	CGTCTTTGCTCTCCATGGAA
CmSWEET1-Q-R	AATGGCTGCAGCGAAACC
CmSWEET2-Q-F	GCTGGGAGTTTTTGGCCTATT
CmSWEET2-Q-R	TTCAAGGTCTGTTACAAGCATGCT
CmSWEET3-Q-F	GTTGCGAATGGCTGTAGGACTA
CmSWEET3-Q-R	TGGGAGCAGCATAAAGCATAA
CmSWEET4-Q-F	AATGCCATTTGTTCACCCAA
CmSWEET4-Q-R	GGCCCCAGATCCATTGGTA
CmSWEET9-Q-F	TGTTGGATTCGGATTGATCGT
CmSWEET9-Q-R	CGATGCACGCCTTTTGC
CmSWEET10-Q-F	TTGTCGGATTTGGATTGATTGTT
CmSWEET10-Q-R	CACACCACGGGTAACTCCACTT
CmSWEET11-Q-F	TCTGATATTCTTCCGGCTGTCA
CmSWEET11-Q-R	GGCTATGTGGCCATCATCTTG
CmSWEET16-Q-F	AGCGTAGAGTACATGCCGTTTCT
CmSWEET16-Q-R	TCCAAACCCCGCCATTAA
CmSWEET17-Q-F	TTGGTGGTGTTGTAGCGGTG
CmSWEET17-Q-R	TATCAGAAGTCGTTCGCTACCAT
